# MMS22L Expression as a Predictive Biomarker for the Efficacy of Neoadjuvant Chemoradiotherapy in Oesophageal Squamous Cell Carcinoma

**DOI:** 10.3389/fonc.2021.711642

**Published:** 2021-09-30

**Authors:** Qiyu Luo, Wenwu He, Tianqin Mao, Xuefeng Leng, Hong Wu, Wen Li, Xuyang Deng, Tingci Zhao, Ming Shi, Chuan Xu, Yongtao Han

**Affiliations:** ^1^ School of Medicine, University of Electronic Science and Technology of China (UESTC), Chengdu, China; ^2^ Department of Thoracic Surgery, Second Affiliated Hospital of Chengdu Medical College (China National Nuclear Corporation 416 Hospital), Chengdu, China; ^3^ Department of Thoracic Surgery, Sichuan Cancer Hospital & Research Institute, School of Medicine, University of Electronic Science and Technology of China (UESTC), Chengdu, China; ^4^ Integrative Cancer Center & Cancer Clinical Research Center, Sichuan Cancer Hospital & Institute Sichuan Cancer Center, School of Medicine, University of Electronic Science and Technology of China, Chengdu, China; ^5^ Department of International Medical Center/Ward of General Practice, West China Hospital, Sichuan University, Chengdu, China; ^6^ Department of Pathology, Sichuan Cancer Hospital & Research Institute, School of Medicine, University of Electronic Science and Technology of China (UESTC), Chengdu, China

**Keywords:** oesophageal squamous cell carcinoma, *MMS22L*, bioinformatics analysis, neoadjuvant chemoradiotherapy, lymph node metastasis, migration

## Abstract

Long-term survival in oesophageal squamous cell carcinoma (ESCC) is related with pathological response after neoadjuvant chemoradiotherapy (NCRT) followed by surgery. However, effective biomarkers to predict the pathologic response are still lacking. Therefore, a systematic analysis focusing on genes associated with the efficacy of chemoradiotherapy in ESCC will provide valuable insights into the regulation of molecular processes. By screening publications deposited in PubMed, we collected genes associated with the efficacy of chemoradiotherapy. A specific subnetwork was constructed using the Steiner minimum tree algorithm. Survival analysis in Kaplan-Meier Plotter online resources was performed to explore the relationship between gene mRNA expression and the prognosis of patients with ESCC. Quantitative real-time polymerase chain reaction (qRT-PCR), Western blotting, and immunohistochemical staining (IHC) were used to evaluate the expression of key genes in cell lines and human samples. The areas under the receiver operating characteristic (ROC) curves (AUCs) were used to describe performance and accuracy. Transwell assays assessed cell migration, and cell viability was detected using the Cytotoxicity Assay. Finally, we identified 101 genes associated with efficacy of chemoradiotherapy. Additionally, specific molecular networks included some potential related genes, such as *CUL3*, *MUC13*, *MMS22L*, *MME*, *UBC*, *VAPA*, *CYP1B1*, and *UGDH.* The *MMS22L* mRNA expression level showed the most significant association with the ESCC patient outcome (*p* < 0.01). Furthermore, *MMS22L* was downregulated at both the mRNA (*p* < 0.001) and protein levels in tumour tissues compared with that in normal tissues. Lymph node metastasis was significantly associated with low *MMS22L* expression (*p* < 0.01). *MMS22L* levels were inversely correlated with the NCRT response in ESCC (*p* < 0.01). The resulting area under the ROC curve was 0.847 (95% CI: 0.7232 to 0.9703; *p* < 0.01). In conclusion, low expression of *MMS22L* is associated with poor response to NCRT, worse survival, lymph node metastasis, and enhanced migration of tumour cells in ESCC.

## Introduction

According to the 2018 Global Cancer Statistics report, oesophageal cancer is among the 10 most frequent carcinomas globally (1). Squamous cell carcinoma (SCC) and adenocarcinoma are the major histologic types of oesophageal carcinoma, and SCC is the main histological type in China (1, 2). Despite advances in surgery, radiotherapy, and chemotherapy, the 5-year survival rate for patients with oesophageal squamous cell carcinoma (ESCC) remains markedly poor because of an advanced stage at diagnosis, the presence of tumour heterogeneity, and insufficient tumour prognostic factors (3, 4). Regarding ESCC at moderate-to-advanced stages, both effective preoperative chemotherapy and chemoradiotherapy have been widely used to shrink tumour size, repress tumour growth or metastasis, increase the R0 resection rate, and reduce the local recurrence rate of ESCC, to improve overall survival compared with surgery alone (5–8). No significant benefits were found in patients who did not respond to the conventional therapy because of toxicity from neoadjuvant chemoradiation therapy (NCRT); they might miss the best timing of treatment and obtain worse prognosis (9–11).

Tumour heterogeneity presents a challenge to successfully treat cancer using chemoradiotherapy, and it is a major factor in chemoradiotherapy failure (12, 13). To enable individualised treatment, screening out response cases and avoiding overtreatment of patients who would not benefit from the inclusion of chemotherapy with sensitive biomarkers are critical. In recent years, although some studies have attempted to reveal the biomarkers that predict responses to chemoradiotherapy, no reliable biomarkers have been identified to assess the efficacy of NCRT in ESCC. Therefore, large amount of studies are needed to further refine the biomarkers for easy use and validate them as biomarkers for future clinical trials.

Identifying new biomarkers is warranted to assist in screening patients who can benefit from chemotherapy and chemoradiotherapy based on bioinformatics analysis, offering consolidated validation for the individual candidate genes. In this study, we collected genes potentially associated with efficacy of chemoradiotherapy to infer specific molecular networks associated with the efficacy of chemoradiotherapy, and some potential related genes were identified. Additionally, the primary aim of the study was to measure potential related gene expression levels in ESCC tissues and evaluate their value as potential predictive biomarkers for the NCRT response in ESCC.

## Materials and Methods

### Candidate Gene Set Approach

As reported previously (4), the efficacy of chemoradiotherapy in patients with ESCC is associated with multiple genes. In the present study, all the genes were obtained by systematic analysis of the human genetic association studies deposited in PubMed (https://www.ncbi.nlm.nih.gov/pubmed). Briefly, with reference to published studies, the search terms were: “(chemotherapy OR radiotherapy OR chemoradiotherapy) AND (cancer OR carcinoma OR neoplasm OR tumour) AND (esophagus OR gastroesophageal) AND (genetic polymorphism OR genes)”, and the date of the last search was September 15, 2019. In total, 217 abstracts were identified by the final electronic searches in PubMed. We included abstracts with sufficient evaluation data, including the methodology, the definition of outcomes, and an appropriate evaluation matrix. Studies without any kind of validation (external validation or internal validation) were excluded. We excluded reviews, editorials, nonhuman studies, and letters without sufficient data. In total, 138 studies were excluded because they failed to meet the above criteria, and 79 articles met the prespecified inclusion criteria. The publications used and the discarded publications can be found in the supplementary documentation (**Supplementary Table S1**). Two reviewers (QL and MT) independently screened the full text and extracted the following information from each study: patient race, number of positive cases, interventions, histological type, the origin of specimens, and genes. Finally, we assembled the purpose genes associated with the response to chemoradiotherapy in ESCC histological type or cell lines.

### Functional Enrichment Analysis

Gene Ontology (GO) and Kyoto Encyclopedia of Genes and Genomes (KEGG) functional enrichment analyses were performed using the R package “cluster Profiler” (14). For the GO and pathway enrichment analysis results, the *p*-value and *q*-values were calculated using Fisher’s exact test and the R package (*p*-value <0.05 and *q*-value <0.05).

### Construction of the Protein Subnetwork

In the context of a human protein-protein interaction (PPI) data set obtained from Protein Interaction Network Analysis platform (PINA) (15), we applied the Steiner minimum tree algorithm implemented in our software framework GenRev to construct a specific subnetwork by using the candidate gene set as seeds (16). A PPI network was built using the Search Tool for the Retrieval of Interacting Genes (STRING) database (version 11.0; https://string-db.org/) (17) and visualised by Cytoscape (an open-source software platform) (18).

### Survival Analysis of New Genes Based on mRNA Expression

The prognostic value of the expression of new gene mRNAs was evaluated using publicly available miRNA expression datasets (Pan-cancer RNA-seq) in Kaplan-Meier plotter (http://kmplot.com/analysis/), an online database including gene expression data and clinical data (19, 20). To assess the prognostic value of a specific gene, the patient samples were divided into two cohorts (high and low expression groups) according to the median mRNA expression of the gene. We analysed the overall survival (OS) of ESCC patients by using a Kaplan-Meier survival plot. Briefly, eight genes (*CUL3*, *MUC13*, *MMS22L*, *MME*, *UBC*, *VAPA*, *CYP1B1*, and *UGDH*) were uploaded into the database respectively to obtain the Kaplan-Meier survival plots, in which the number-at-risk is shown below the main plot. Log rank *p*-value and hazard ratio (HR) with 95% confidence intervals were calculated and displayed on the picture. A *p*-value <0.05 was considered statistically significant.

### Patients and Tumour Samples

Sixty-one ESCC tissues were obtained from the Department of Thoracic Surgery of Sichuan Cancer Hospital (Chengdu, China) from Jan 2018 to Sept 2019 in this study, and all the samples were histologically confirmed to be ESCC tissues by a postoperative pathologist. Twenty-three paired human ESCC cancer tissues and matched adjacent normal tissues (located at least 5 cm from the tumour border, **Supplementary Figure S1**) from 23 patients with surgery alone were snap frozen after they were taken from surgery and then stored in liquid nitrogen for quantitative real-time PCR and Western blotting. Another 38 ESCC biopsy specimens from the gastroscopies were paraffin embedded for immunohistochemical staining analysis before patients received NCRT. The samples used in the study were approved by the Ethical Committee of the Sichuan Cancer Hospital (No. SCCHEC-02-2017-043), and the patients provided written informed consent to participants.

### Cell Culture

The human ESCC cell lines TE-1, Kyse150, and Eca109 and the human normal oesophageal cell line HEEC were provided by the Shanghai Institute of Cell Biology, Chinese Academy of Sciences. All the cell lines were maintained in RPMI-1640 (HyClone, USA) supplemented with 100 U/ml of penicillin-streptomycin (HyClone, USA) and 10% foetal bovine serum (FBS; Gibco, USA) at 37°C with 5% CO_2_ in a humidified atmosphere.

### Quantitative Real-Time PCR

The mRNA expression of *MMS22L* was measured by quantitative real-time polymerase chain reaction (qRT-PCR). The above cell clines and 23 paired human ESCC cancer tissues and matched adjacent normal tissues were individually homogenised in liquid nitrogen, and total RNA was extracted from cells and tissues using TRIzol reagent (Invitrogen, Waltham, MA, USA). The total RNA products were immediately transcribed into cDNA using a cDNA synthesis kit (Takara, Kyoto, Japan) following the manufacturer’s instructions. Complementary DNA was amplified using TB Green™ Advantage^®^ qPCR Premix (Takara, Kyoto, Japan) on the CFX-Connect Real-Time PCR Detection System (Bio-Rad, Hercules, CA, USA). The *MMS22L* primers were as follows: forward 5′-CAGAGAATGTCACAGGTAGTGCC-3′; reverse 5′-TCTGATGGAGCTGTGCTTGGCA-3′. The conditions for qRT-PCR were as follows: initial denaturation at 95°C for 2 min, followed by 40 cycles of 95°C for 5 s and 55°C–57°C for 30 s, and melting curves were generated by heating from 55°C to 95°C with 0.5°C increments each cycle. The results were normalised to *β*-actin using the 2^−ΔΔCt^ method (21), the forward primer: 5′-CTTAGTTGCGTTACACCCTTTCTTG-3′ and reverse primer 3′-ACTGCTGTCACCTTCACCGTTC-5′. All the experiments were performed in triplicate.

### Western Blotting

The Total proteins of tissues and cells were extracted using RIPA buffer with protease inhibitors (Biyuntian, China). The protein concentrations were measured using the bicinchoninic acid (BCA) Protein Assay Kit (Beijing Suolabao Biotech, China). The *MMS22L* protein levels in cancer tissue and adjacent tissue were evaluated by Western blotting (WB). Briefly, equal amounts of total protein extract were first separated in an 8% SDS-PAGE gel and transferred onto PVDF membranes (Millipore, Billerica, MA, USA). Next, the membranes were blocked for 2 h with PBST and 5% milk at room temperature, incubated with primary antibody overnight at 4°C and then with secondary antibody for 2 h at room temperature. Protein expression was detected using an anti-*MMS22L* (*C6orf167*) antibody (ab181047, 1:1,000, Abcam, Cambridge, MA, USA) and a *β*-actin polyclonal antibody (1:1,000 CST, Danvers, MA, USA). Protein bands were detected using an Immobilon^®^ Western system (Millipore; #WBKLS0100) and imaged on the Minichemi machine (Sage Creation, Beijing, China).

### Immunohistochemical Staining and Image Analysis

Tissues from paraffin-embedded blocks were sectioned at 5-μm thickness. Immunohistochemical staining (IHC) was performed using DAB kit (Zhongshan Golden Bridge, Beijing, China) following the manufacturer’s protocol. The anti-*MMS22L* antibody (bs17689R) used for IHC was purchased from Bioss (Beijing, China). To quantify *MMS22L* staining, five randomly chosen fields per section were evaluated at ×200 magnification for each sample. Image-Pro Plus 6.0 was used to determine integrated optical density (IOD) values, and the IOD per unit area (mean density) represented the relative *MMS22L* expression level.

### Cell Transfection

Two target small hairpin RNA (shRNA) lentiviruses of the *MMS22L* gene, sh147 and sh148 and a negative control shRNA lentivirus con077 were designed and synthesised by Shanghai Ji Kai Gene Technology Co., Ltd. (Shanghai, China). TE-1 cells were infected with lentivirus at an MOI of 10 PFU per cell. Stable transformants were selected with 2 μg/ml of puromycin. The knockdown efficiency was detected by qRT-PCR and Western blotting.

### Transwell Assay

Transwell migration assays were performed using plates (Corning, Corning, NY, USA) with 8‐μm‐pore size membranes. Briefly, 2 × 10^4^ cells were suspended in 200 μl of FBS‐free RPMI‐1640 medium and added to the upper chambers of Transwell plates. RPMI‐1640 medium (500 μl) supplemented with 5% FBS was seeded into the lower chamber. After a 24‐h incubation period at 37°C and 5% CO_2_, the migrated cells were fixed with 4% paraformaldehyde (in 1 × PBS) for 20 min at room temperature and then were stained with crystal violet.

### Cytotoxicity Assay

Cytotoxicity was assayed using cell counting kit (CCK8; Hanheng, Shanghai, China) following the manufacturer’s instructions. Briefly, cells were seeded on 96-well plates at a density of 2.0 × 10^3^ per well. After cells were treated with 5-FU (Selleck, Houston, TX, USA) at concentrations of 0, 0.1, 1, 5, 10, and 100 μM for 72 h, the medium containing 5-FU was exchanged for 100 μl of RPMI-1640, and 10 μl of CCK8 reagent was added. Two hours later, absorbance at 450 nm was measured using a microplate reader. Each group had three repeats, and the experiment was repeated three times.

### Statistical Analysis

All the data were obtained from at least three independent measurements and were shown as means ± SEM. Student’s *t*-test, Mann-Whitney tests, and receiver and IC_50_ operating characteristic (ROC) curves were performed using GraphPad Prism 7 software (GraphPad Software Inc., San Diego, CA, USA). One-way analysis of variance (ANOVA) followed by Tukey’s *post-hoc* test was used for data analysis among three or more groups. Statistical analysis was conducted, and statistical significance was set at *p* < 0.05.

## Results

### Candidate Gene Sets and Functional Enrichment

**Figure 1:**
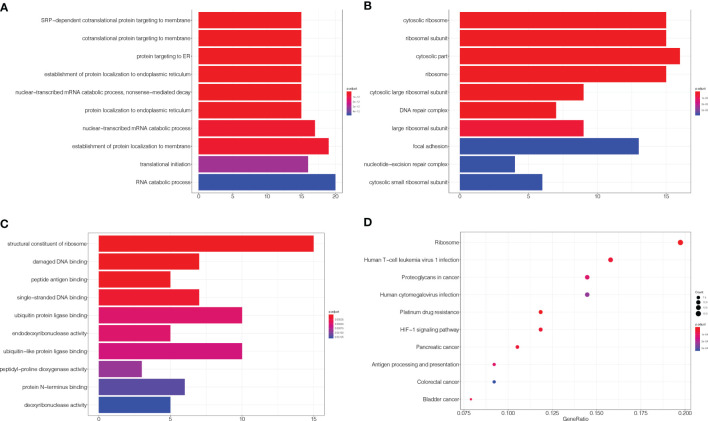
GO and KEGG enrichment of related genes. **(A–C)** The top 10 GO terms in biological process, cellular component, and molecular function, respectively. **(D)** KEGG pathways were analysed, and the top 10 pathways are shown. The *x*-axis shows the GeneRatio, whereby a higher value indicates more genes enriched in the pathway; the *y*-axis shows the enriched pathways, and the redder the dot is, the more significant is the pathway.

As described in the *Materials and Methods* section, 101 candidate genes associated with the efficacy of chemoradiotherapy in patients with ESCC were assembled after removing duplicates. The GO annotations were classified as biological process, cellular component, and molecular function (*p* < 0.05), and the top 10 GO terms are shown in **Figures 1A–C**, respectively. The top biological process GO enrichment terms were cotranslational protein targeting the membrane, targeting the ER, and establishment of protein localisation to endoplasmic reticulum. Ribosome component was the most enriched cellular component GO term. Additionally, we found several enriched molecular functions such as structural constituent of ribosome, damaged DNA binding, antigen binding, single-stranded DNA binding, and ubiquitin protein ligase binding. Furthermore, the KEGG pathways in which 101 candidate genes were mostly enriched were ribosome, followed by human T-cell leukaemia virus 1 infection, platinum drug resistance, human cytomegalovirus infection, proteoglycans in cancer, etc. (**Figure 1D**).

### Construction of the Protein Subnetwork

**Figure 2:**
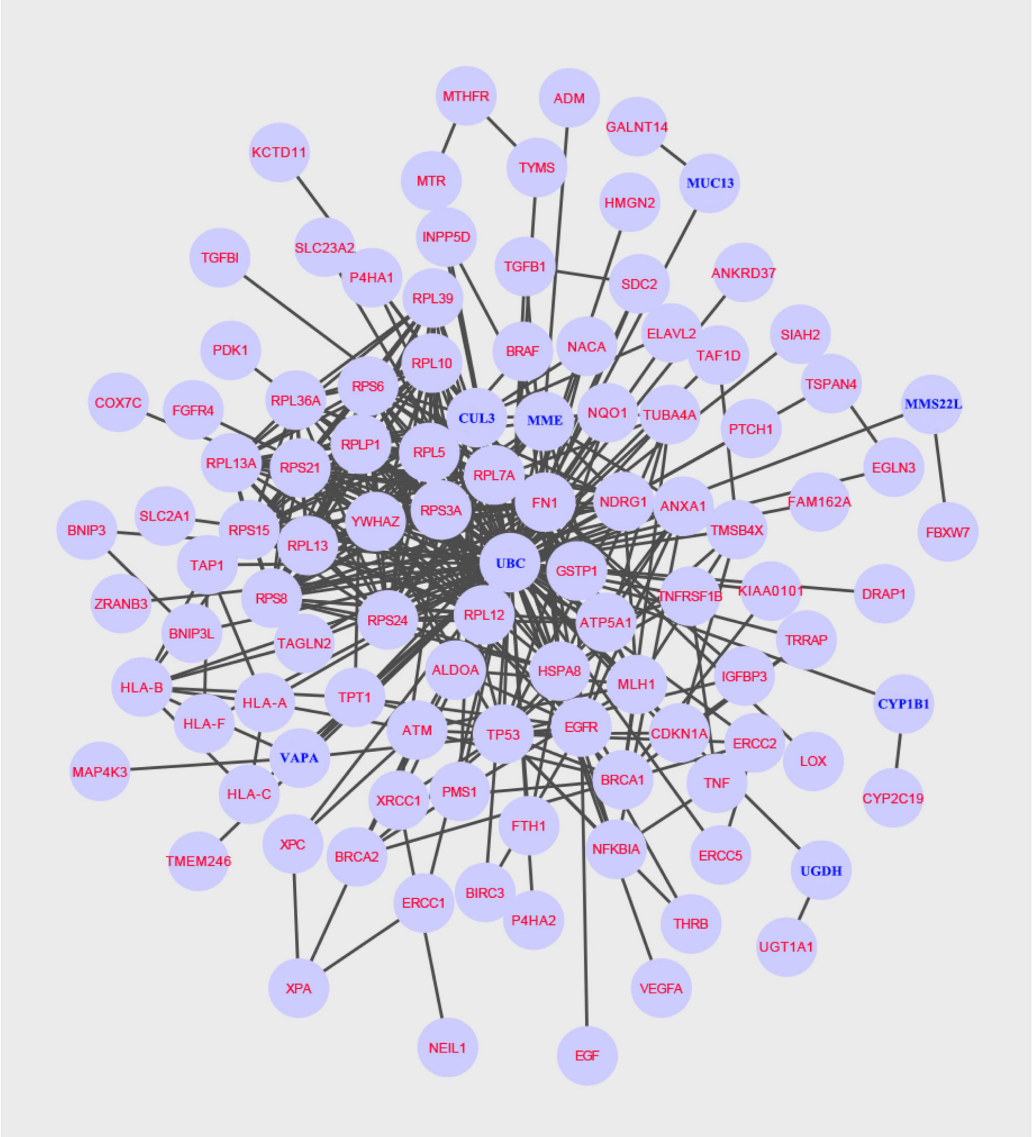
A specific protein network associated with NCRT efficacy is built using the Steiner minimal tree algorithm, including 105 nodes and 379 edges. Additionally, eight new genes are labelled in blue.

The protein subnetwork comprised 105 nodes and 379 edges (interactions), and 97 of 101 candidate genes were included in the subnetwork, accounting for 92.38% of 105 genes in the network and 96% of 101 candidate genes, and demonstrating a high coverage of the candidate genes set in the subnetwork (**Figure 2**). Additionally, eight genes in the subnetwork outside of the candidate gene set were obtained (**Table 1**).

**Table 1:** Identification of eight potentially related genes associated with chemoradiotherapy efficacy: *UGDH*, *VAPA*, *MME*, *CUL3*, *UBC*, *CYP1B1*, *MUC13*, and *MMS22L*.

Gene symbol	Gene ID	Map location	Description
*UGDH*	7358	4p14	UDP-glucose 6-dehydrogenase
*MME*	4311	3q25.2	Membrane metalloendopeptidase
*VAPA*	9218	18p11.22	VAMP-associated protein A
*CUL3*	8452	2q36.2	Cullin 3
*UBC*	7316	12q24.31	Ubiquitin C
*CYP1B1*	1545	2p22.2	Cytochrome P450 family 1 subfamily B member 1
*MUC13*	56667	3q21.2	Mucin 13, cell surface associated
*MMS22L*	253714	6q16.1	MMS22 like, DNA repair protein

### Prognostic Value of mRNA Expression of Eight New Genes in ESCC

**Figure 3:**
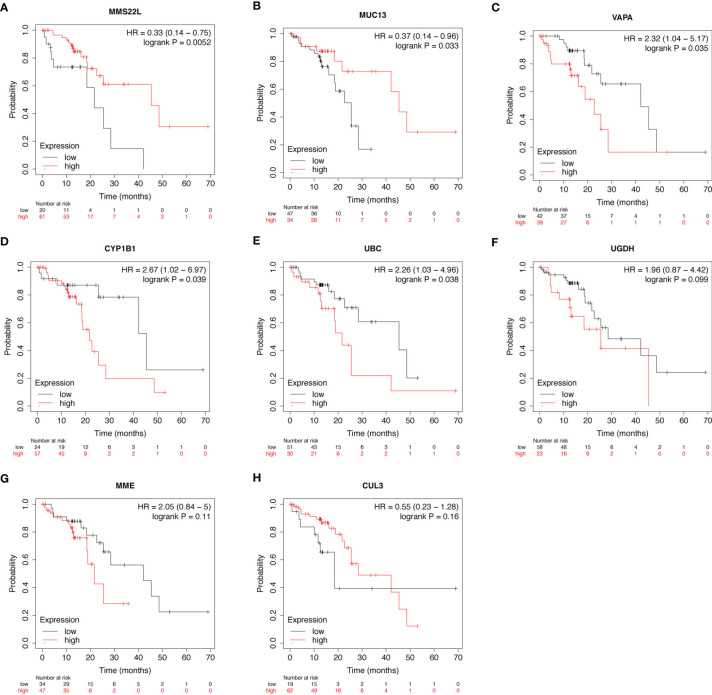
Prognostic value of mRNA expression of eight genes in patients with ESCC. **(A–H)** Overall Survival curves of MMS22L **(A)**, MUC13 **(B)**, VAPA **(C)**, CYP1B1 **(D)**, UBC **(E)**, UGDH **(F)**, MME **(G)**, and CUL13 **(H)** were plotted in Kaplan-Meier plotter. “Probability” on the y-axis represents the survival rates, the red line represents the patients with mRNA expression above the median, and the black line represents the patients with mRNA expression below the median. ESCC, Oesophageal squamous cell carcinoma; HR, hazard ratio.

Using the Kaplan-Meier plotter database, the prognostic value of the eight novel genes was evaluated in ESCC patients. We found that higher and lower expression of five biomarkers was significantly associated with overall survival. ESCC patients with higher mRNA levels of *MMS22L* (HR = 0.33; CI: 0.14−0.75; logrank *p* = 0.0052) and *MUC13* (HR = 0.37; CI: 0.14−0.96; logrank *p* = 0.033) had higher OS (**Figures 3A, B** **)**, and ESCC patients with lower mRNA levels of *VAPA* (HR = 2.32; CI: 1.04−5.17; logrank *p* = 0.035), *CYP1B1* (HR = 2.67; CI: 1.02−6.97; logrank *p* = 0.039), and *UBC* (HR = 2.26; CI: 1.03−4.96; logrank *p* = 0.038) had higher OS (**Figures 3C–E**), while the mRNA expression of *UGDH, MME* and *CUL13* were not associated with ESCC patient survival (**Figures 3F–H**).

### Expression of the *MMS22L* Genes in Cancer and Adjacent Normal Tissues

**Figure 4:**
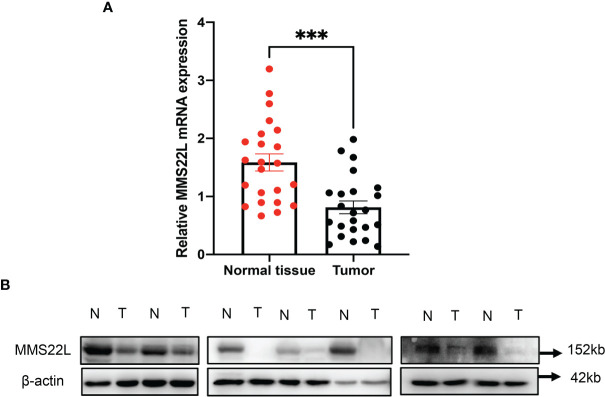
The *MMS22L* mRNA and protein levels are significantly lower in cancerous tissues than in normal adjacent tissues. **(A)** *MMS22L* mRNA was detected by qRT-PCR, and relative quantification analysis was normalised to *β-actin* mRNA (***p < 0.001). **(B)** *MMS22L* protein expression was detected in randomly selected seven ESCC tissues and adjacent normal tissues using Western blotting. T, tumour tissues; N, normal adjacent tissues.

*MMS22L* is not a well-studied protein, and information about this gene product is very limited. As described above, the expression of *MMS22L* had the most significant association with ESCC patient outcome. Cellular components associated with the function of *MMS22L* were significantly enriched, such as damaged DNA binding and ubiquitin protein ligase binding. Therefore, we further detected the expression level of the *MMS22L* gene in ESCC tissues and adjacent normal tissues using quantitative real-time PCR and WB. MM*S22L* mRNA expression was significantly decreased in ESCC tissue compared with that in adjacent normal tissues (*p* < 0.05) (**Figure 4A**), and WB results were consistent with the RT-qPCR (**Figure 4B**). *MMS22L* protein expression was significantly reduced in seven tumour tissues compared with that in normal adjacent tissues.

### Predictive Value of *MMS22L* for NCRT Efficacy in Patients With ESCC

**Figure 5:**
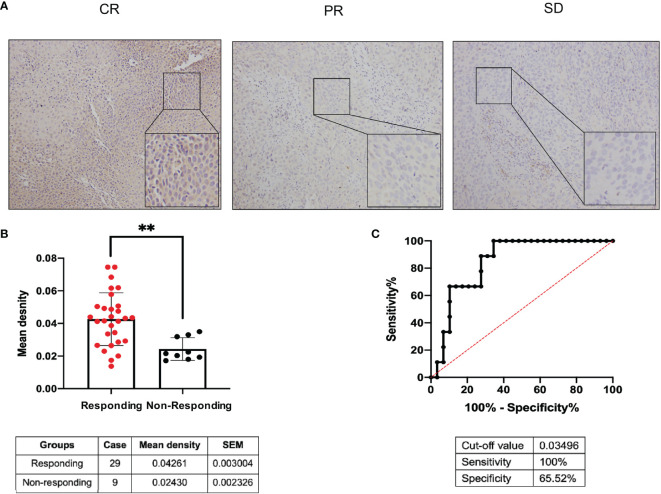
Predictive value of *MMS22L* for different responses to NCRT in patients with ESCC. **(A)** *MMS22L* IHC in representative tumour tissues. **(B)** The mean density of the responding group and nonresponding group from all 38 cases who received NCRT is summarised in the table below. **(C)** ROC curve for evaluating the predictive value of *MMS22L* for ESCC patients with NCRT. The AUC was 0.847, the sensitivity was 100%, the specificity was 65.52%, and the cutoff value was 0.03495 (**p < 0.01). NCRT, neoadjuvant chemoradiotherapy; AUC, area under the ROC curve; ROC, receiver operating characteristic.

According to the RECIST 1.1 criteria (22), the response to NCRT was defined as complete response (CR), partial response (PR), stable disease, (SD), and progressive disease (PD), and the 38 patients receiving NCRT were divided into the responding group (CR plus PR, 29 patients) and nonresponding group (SD, nine patients) according to the response to NCRT in this study. *MMS22L* IHC in representative tumour tissues are shown in **Figure 5A**. The *MMS22L* protein in the responding group was significantly higher than that in the nonresponding groups (*p* < 0.01, **Figure 5B**). This result indicated that the *MMS22L* expression significantly predicted a response to NACRT. ROC curve analyses revealed that *MMS22L* was a valuable biomarker for differentiating responding from nonresponding for NCRT. *MMS22L* yielded an AUC of 0.847 (95% CI: 0.7232 to 0.9703; *p* <  0.01; **Figure 5C**) with 100% sensitivity and 65.52% specificity in predicting the efficacy of NCRT in ESCC. Additionally, among the various clinicopathological characteristics evaluated, no significant associations were found between the *MMS22L* expression level and age, sex, differentiation, location, depth of invasion, or TNM clinical stage, but a low expression level of *MMS22L* was markedly associated with lymph node metastasis (*p* < 0.01; **Table 2**).

**Table 2:** Association of *MMS22L* expression in the biopsy specimens with clinicopathological parameters.

Variable	*N*	Mean density ± SEM	*p*-Value
Age (year)			
≥60	24	0.03872 ± 0.003568	0.9366
<60	14	0.03698 ± 0.003682	
Sex			
Male	33	0.03859 ± 0.002842	0.7376
Female	5	0.03618 ± 0.008554	
Differentiation			
G1/Gx	24	0.04380 ± 0.003507	0.2028
G2	6	0.04526 ± 0.01146	
G3	8	0.02946 ± 0.004677	
Location			
Upper	8	0.03477 ± 0.005017	0.8406
Moderate	19	0.03978 ± 0.004123	
Lower	11	0.03901 ± 0.005607	
Depth of invasion			
cT1-T2	13	0.04069 ± 0.003240	0.2587
cT3-T4	25	0.03790 ± 0.003948	
Lymph node metastasis			
cN0	27	0.04188 ± 0.002912	0.0095
cN+	11	0.02942 ± 0.005090	
Stage			
cI-II	13	0.03962 ± 0.003136	0.3449
cIII-IV	25	0.03757 ± 0.003753	

The mean density represents the relative MMS22L expression level.

### *MMS22L* Inhibits Migration and Modulates 5-FU Sensitivity in TE-1 Cells

**Figure 6:**
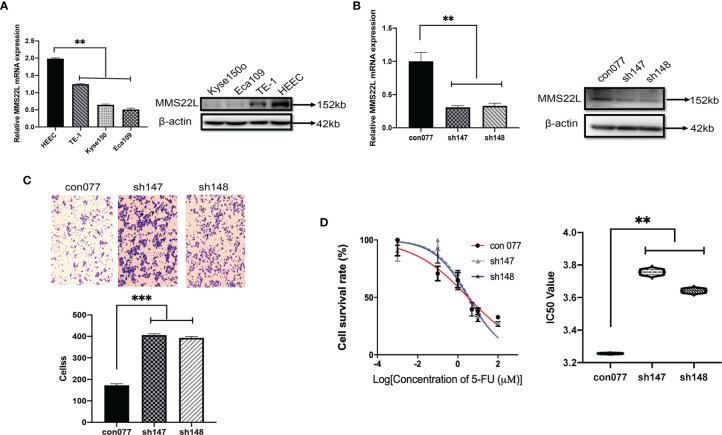
Lower expression of *MMS22L* increases the migration capabilities and the 5-FU resistance of TE-1 cells. **(A)** *MMS22L* expression in a human normal oesophageal cell line (HEEC) and human ESCC cell lines. **(B)** The transfection efficiency was measured after 48 h. **(C)** In the shRNA groups, the migration capabilities increased and the number of migrating cells increased compared with those in the con077 group. **(D)** IC_50_ values of TE-1 cells to 5-FU were detected *via* the CCK8 assay after transfection (**p < 0.01; ***p < 0.001). CCK8, cell counting kit; IC_50_, half-maximal inhibitory concentration; 5-FU, 5-fluorouracil.

To assess *MMS22L* expression in human ESCC cells, we detected the mRNA and protein expression of *MMS22L* in HEEC cells and three human ESCC cell lines (TE-1, Kyse150, and Eca109) by qRT-PCR and WB, respectively. The expression of *MMS22L* was lower in TE-1, Kyse150, and Eca109 cells than in HEEC cells (**Figure 6A**). TE-1 cells showed the highest *MMS22L* expression in three human ESCC cell lines. Thus, TE-1 cells were selected for subsequent assays. After targeting *MMS22L* with two different shRNAs, the knockdown efficiency was detected (**Figure 6B**). We performed Transwell migration assays to analyse whether *MMS22L* inhibits the migration ability of TE-1 cells. *MMS22L* inhibited the migration of TE-1 cells (**Figure 6C**). Different TE-1 cells were determined by CCK8 assays with different doses of 5-FU to calculate the IC_50_ and were performed in triplicate. The IC_50_ value decreased significantly after *MMS22L* knockout (**Figure 6D**). These results from *in vitro* experiments indicated that the knockdown of *MMS22L* in the TE-1 cell line is associated with enhanced migration and resistance to 5-FU.

## Discussion

According to published statistics, approximately 572,034 new oesophageal cancer cases (3.2%) and an estimated 508,585 related deaths (5.3%) occurred (1). With the development of comprehensive treatment strategies, patients with ESCC benefit from NCRT (5). However, the postoperative pathological complete response rate was only 43.2% according to a phase III, multicentre, randomised open-label clinical trial (NEOCRTEC5010) (23). Therefore, further understanding of NCRT resistance-related genes as novel prognostic biomarkers in ESCC is necessary. Recently, an increasing number of potential genes have been found to be associated with chemoradiotherapy efficacy and prognosis in patients with cancer. However, a few of these predicted genes have been identified in ESCC, and a few biomarkers are available for clinical monitoring.

In our study, we applied the Steiner minimum tree algorithm to explore novel biomarkers in the context of a human PPI background. First, a list of 101 candidate genes associated with the efficacy of chemoradiotherapy in patients with ESCC was assembled in 79 relevant articles. Simultaneously, to better understand the function of these genes in ESCC, we performed GO and KEGG analyses. Notably, KEGG analysis revealed that the pathways in which these genes are mainly enriched are ribosome, human T-cell leukaemia virus 1 infection, platinum drug resistance, human cytomegalovirus infection, and proteoglycans in cancer. Second, we obtained eight novel genes associated with linking genes potentially related to chemoradiotherapy efficacy in ESCC outside the candidate gene set. Referring to published studies, the findings of our study validate previous reported outcomes because different functions of these genes have been identified to be associated with ESCC. *MUC13* is a potential biomarker to predict the efficacy of neoadjuvant chemotherapy in ESCC patients (24, 25). Moghadam et al. suggest a relationship between the *CYP1B1*-rs1056836 genetic polymorphism and clinical features of ESCC (26). Therefore, the results of the present study are considered to be reliable.

To further identify effective biomarkers with diagnostic and prognostic value, we evaluated the effects of the eight novel genes on the survival of ESCC patients using the online Kaplan-Meier plotter. ESCC patients with higher mRNA levels of *MMS22L* and *MUC13* had higher OS. Additionally, ESCC patients with lower mRNA levels of *VAPA*, *CYP1B1*, and *UBC* had higher OS. As shown in the Kaplan-Meier plotter, *MMS22L* expression was the most likely candidate gene among many novel genes. The *MMS22L* gene is mapped to chromosome 6 open-reading frame 167, also known as *C6orf167*. In our study, *MMS22L* expression in 23 ESCC tissues was notably lower than that in their para-carcinoma tissues, and this trend was consistently observed across the ESCC cell lines and the human normal oesophageal cell line. Thus, *MMS22L* may be a tumour suppressor gene in ESCC. In contrast to our findings, a previous study found that *MMS22L* expression was upregulated in lung and oesophageal cancer tissues compared with that in adjacent normal lung and oesophageal tissues, and *MMS22L* was identified as an oncogene (27). This discrepancy may arise from the inadequate sample size and unknown oesophageal cancer histologic type in their study. Many genes play both tumour suppressor or oncogenic roles in different tissues, tumour types, or cellular contexts (28).

Previous studies have shown that *MMS22L* plays critical roles in the DNA damage response and repair, affecting the response of tumour cells to DNA-damaging agents, such as camptothecin (CPT), ionising radiation (IR), hydroxyurea (HU), and methyl methanesulfonate (MMS) (29–32). According to previous reports, knocking down *MMS22L* promoted apoptosis by activating caspase-3 and downregulating *Bcl-XL* (29), an inhibitor of apoptosis. Importantly, Duro et al. found that tumour cells lacking *MMS22L* were resistant to HU, cisplatin, and IR. However, this finding is inconsistent with that reported by O’Connell et al. who found that Hela cells lacking *MMS22L* were sensitive to IR, CPT, and MMS (30). Additionally, downregulation of *MMS22L* is associated the bone metastasis in breast cancer (33). Previous controversial results have generated considerable interest regarding the function of *MMS22L* in ESCC. In the present study, we found that low *MMS22L* expression, immunohistochemically stained and analysed semiquantitatively in ESCC samples, was a useful predictor of worse responses to NCRT and lymph node metastasis, and enhanced migration and resistance to 5-FU in the TE-1 cell line. These findings were corroborated by *in vitro* studies showing that the knockdown of *MMS22L* in the TE-1 cell line was associated with enhanced migration and resistance to 5-FU.

Proteins associated with each other in the String Protein-Protein Interaction Network have related functions, and *FBXW7* is most closely related to *MMS22L*. *FBXW7* is an F-box protein that binds to key regulators of cell division and growth, including cyclin E, *MYC*, *JUN*, and Notch (34). *FBXW7* is a critical tumour suppressor involved in the ubiquitin-proteasome system in human cancer, and loss of *FBXW7* function leads to chromosomal instability (34, 35). *FBXW7* expression is downregulated in ESCC tissues and correlates with the TNM stage, the degree of differentiation, the invasion depth, the lymph node metastasis, and a worse prognosis in ESCC (36, 37). Mutations in *FBXW7* are associated with metastasis and correlates with increased expression of T-cell proliferation and antigen presentation functions (38). These results may suggest that an indirect or direct regulatory function between *MMS22L* and *FBXW7* will contribute to tumour progression and metastasis through immunological effects or the tumour microenvironment. However, this interpretation of these data remains speculative at this point and will require further cell biological studies.

In conclusion, network analysis based on molecular aspects associated with the efficacy of chemoradiotherapy in ESCC may facilitate identification of novel biomarkers and a deeper understanding of the mechanisms. Our approach presents interesting approaches for future studies. Additionally, our data indicate that low *MMS22L* expression status in biopsy specimens is a predictive factor for the unfavourable efficacy of NACRT in ESCC. Therefore, patients with ESCC receiving NCRT are likely to fail when *MMS22L* downregulation is observed on a biopsy specimen. However, a larger sample size will be needed to validate and possibly extend these findings.

## Data Availability Statement

The original contributions presented in the study are included in the article/**Supplementary Material**. Further inquiries can be directed to the corresponding authors.

## Ethics Statement

The studies involving human participants were reviewed and approved by the Ethical Committee of the Sichuan Cancer Hospital (No. SCCHEC-02-2017-043). The patients/participants and legal guardian/next of kin provided written informed consent to participate in this study.

## Author Contributions

Conception and design: YH and CX. Acquisition of data: QL and WH. Analysis and interpretation of data: TM and XL. Drafting of the manuscript: QL and WH. Critical revision of the manuscript for important intellectual content: YH and CX. Harvesting of the specimen: XD and MS. Administrative, technical, or material support: HW, WL, and TZ. All authors contributed to the article and approved the submitted version.

## Funding

This work was supported by grants from the National Key R&D Program of China (2016YFC0901401), Bethune Charitable Foundation (HZB-20190528-19) and the Applied Basic Research Programs of Science and Technology Commission Foundation of Sichuan Province, China (2020YJ0174).

## Conflict of Interest

The authors declare that the research was conducted in the absence of any commercial or financial relationships that could be construed as a potential conflict of interest.

## Publisher’s Note

All claims expressed in this article are solely those of the authors and do not necessarily represent those of their affiliated organizations, or those of the publisher, the editors and the reviewers. Any product that may be evaluated in this article, or claim that may be made by its manufacturer, is not guaranteed or endorsed by the publisher.

## References

[1] Bray F Ferlay J Soerjomataram I Siegel RL Torre LA Jemal A. Global Cancer Statistics 2018: GLOBOCAN Estimates of Incidence and Mortality Worldwide for 36 Cancers in 185 Countries. CA Cancer J Clin (2018) 68(6):394–424. doi: 10.3322/caac.21492 30207593

[2] Liang H Fan JH Qiao YL. Epidemiology, Etiology, and Prevention of Esophageal Squamous Cell Carcinoma in China. Cancer Biol Med (2017) 14(1):33–41. doi: 10.20892/j.issn.2095-3941.2016.0093 28443201 PMC5365188

[3] Liu M He Z Guo C Xu R Li F Ning T. Effectiveness of Intensive Endoscopic Screening for Esophageal Cancer in China: A Community-Based Study. Am J Epidemiol (2019) 188(4):776–84. doi: 10.1093/aje/kwy291 30608546

[4] Gusella M Pezzolo E Modena Y Barile C Menon D Crepaldi G. Predictive Genetic Markers in Neoadjuvant Chemoradiotherapy for Locally Advanced Esophageal Cancer: A Long Way to Go. Review of the Literature. Pharmacogenomics J (2018) 18(1):14–22. doi: 10.1038/tpj.2017.25 28607505

[5] Shapiro J van Lanschot JJB Hulshof M van Hagen P van Berge Henegouwen MI Wijnhoven BPL. Neoadjuvant Chemoradiotherapy Plus Surgery *Versus* Surgery Alone for Oesophageal or Junctional Cancer (CROSS): Long-Term Results of a Randomised Controlled Trial. Lancet Oncol (2015) 16(9):1090–8. doi: 10.1016/s1470-2045(15)00040-6 26254683

[6] van Hagen P Hulshof MC van Lanschot JJ Steyerberg EW van Berge Henegouwen MI Wijnhoven BP. Preoperative Chemoradiotherapy for Esophageal or Junctional Cancer. New Engl J Med (2012) 366(22):2074–84. doi: 10.1056/NEJMoa1112088 22646630

[7] Meredith KL Weber JM Turaga KK Siegel EM McLoughlin J Hoffe S. Pathologic Response After Neoadjuvant Therapy is the Major Determinant of Survival in Patients With Esophageal Cancer. Ann Surg Oncol (2010) 17(4):1159–67. doi: 10.1245/s10434-009-0862-1 20140529

[8] Oppedijk V van der Gaast A van Lanschot JJ van Hagen P van Os R van Rij CM. Patterns of Recurrence After Surgery Alone *Versus* Preoperative Chemoradiotherapy and Surgery in the CROSS Trials. J Clin oncology: Off J Am Soc Clin Oncol (2014) 32(5):385–91. doi: 10.1200/jco.2013.51.2186 24419108

[9] Dittrick GW Weber JM Shridhar R Hoffe S Melis M Almhanna K. Pathologic Nonresponders After Neoadjuvant Chemoradiation for Esophageal Cancer Demonstrate No Survival Benefit Compared With Patients Treated With Primary Esophagectomy. Ann Surg Oncol (2012) 19(5):1678–84. doi: 10.1245/s10434-011-2078-4 22045465

[10] Donahue JM Nichols FC Li Z Schomas DA Allen MS Cassivi SD. Complete Pathologic Response After Neoadjuvant Chemoradiotherapy for Esophageal Cancer Is Associated With Enhanced Survival. Ann Thorac Surg (2009) 87(2):392–8; discussion 8-9. doi: 10.1016/j.athoracsur.2008.11.001 19161745 PMC2930775

[11] Pasini F de Manzoni G Zanoni A Grandinetti A Capirci C Pavarana M. Neoadjuvant Therapy With Weekly Docetaxel and Cisplatin, 5-Fluorouracil Continuous Infusion, and Concurrent Radiotherapy in Patients With Locally Advanced Esophageal Cancer Produced a High Percentage of Long-Lasting Pathological Complete Response: A Phase 2 Study. Cancer (2013) 119(5):939–45. doi: 10.1002/cncr.27822 23165781

[12] Gerlinger M Rowan AJ Horswell S Math M Larkin J Endesfelder D. Intratumor Heterogeneity and Branched Evolution Revealed by Multiregion Sequencing. New Engl J Med (2012) 366(10):883–92. doi: 10.1056/NEJMoa1113205 PMC4878653 22397650

[13] Wu D Wang DC Cheng Y Qian M Zhang M Shen Q. Roles of Tumor Heterogeneity in the Development of Drug Resistance: A Call for Precision Therapy. Semin Cancer Biol (2017) 42:13–9. doi: 10.1016/j.semcancer.2016.11.006 27840278

[14] Yu G Wang LG Han Y He QY. Clusterprofiler: An R Package for Comparing Biological Themes Among Gene Clusters. Omics (2012) 16(5):284–7. doi: 10.1089/omi.2011.0118 PMC3339379 22455463

[15] Wu J Vallenius T Ovaska K Westermarck J Mäkelä TP Hautaniemi S. Integrated Network Analysis Platform for Protein-Protein Interactions. Nat Methods (2009) 6(1):75–7. doi: 10.1038/nmeth.1282 19079255

[16] Zheng S Zhao Z. Genrev: Exploring Functional Relevance of Genes in Molecular Networks. Genomics (2012) 99(3):183–8. doi: 10.1016/j.ygeno.2011.12.005 PMC3292679 22227021

[17] Szklarczyk D Gable AL Lyon D Junge A Wyder S Huerta-Cepas J. STRING V11: Protein-Protein Association Networks With Increased Coverage, Supporting Functional Discovery in Genome-Wide Experimental Datasets. Nucleic Acids Res (2019) 47(D1):D607–d13. doi: 10.1093/nar/gky1131 PMC6323986 30476243

[18] Wiredja D Bebek G. Identifying Gene Interaction Networks. Methods Mol Biol (2017) 1666:539–56. doi: 10.1007/978-1-4939-7274-6_27 28980264

[19] Madamsetty VS Pal K Dutta SK Wang E Mukhopadhyay D. Targeted Dual Intervention-Oriented Drug-Encapsulated (DIODE) Nanoformulations for Improved Treatment of Pancreatic Cancer. Cancers (Basel) (2020) 12(5):1189–208. doi: 10.3390/cancers12051189 PMC7281578 32397114

[20] Quiñones-Díaz BI Reyes-González JM Sánchez-Guzmán V Conde-Del Moral I Valiyeva F Santiago-Sánchez GS. MicroRNA-18a-5p Suppresses Tumor Growth *via* Targeting Matrix Metalloproteinase-3 in Cisplatin-Resistant Ovarian Cancer. Front Oncol (2020) 10:602670. doi: 10.3389/fonc.2020.602670 33392094 PMC7774672

[21] Livak KJ Schmittgen TD. Analysis of Relative Gene Expression Data Using Real-Time Quantitative PCR and the 2(-Delta Delta C(T)) Method. Methods (2001) 25(4):402–8. doi: 10.1006/meth.2001.1262 11846609

[22] Eisenhauer EA Therasse P Bogaerts J Schwartz LH Sargent D Ford R. New Response Evaluation Criteria in Solid Tumours: Revised RECIST Guideline (Version 1.1). Eur J Cancer (2009) 45(2):228–47. doi: 10.1016/j.ejca.2008.10.026 19097774

[23] Yang H Liu H Chen Y Zhu C Fang W Yu Z. Neoadjuvant Chemoradiotherapy Followed by Surgery *Versus* Surgery Alone for Locally Advanced Squamous Cell Carcinoma of the Esophagus (NEOCRTEC5010): A Phase III Multicenter, Randomized, Open-Label Clinical Trial. J Clin Oncol (2018) 36(27):2796–803. doi: 10.1200/jco.2018.79.1483 PMC6145832 30089078

[24] Shen LY Wang H Dong B Yan WP Lin Y Shi Q. Possible Prediction of the Response of Esophageal Squamous Cell Carcinoma to Neoadjuvant Chemotherapy Based on Gene Expression Profiling. Oncotarget (2016) 7(4):4531–41. doi: 10.18632/oncotarget.6554 PMC4826224 26673820

[25] Wang H Shen L Lin Y Shi Q Yang Y Chen K. The Expression and Prognostic Significance of Mucin 13 and Mucin 20 in Esophageal Squamous Cell Carcinoma. J Cancer Res Ther (2015) 11 Suppl 1:C74–9. doi: 10.4103/0973-1482.163846 26323930

[26] Moghadam AR Mehramiz M Entezari M Aboutalebi H Kohansal F Dadjoo P. A Genetic Polymorphism in the CYP1B1 Gene in Patients With Squamous Cell Carcinoma of the Esophagus: An Iranian Mashhad Cohort Study Recruited Over 10 Years. Pharmacogenomics (2018) 19(6):539–46. doi: 10.2217/pgs-2018-0197 29629838

[27] Nguyen MH Ueda K Nakamura Y Daigo Y. Identification of a Novel Oncogene, MMS22L, Involved in Lung and Esophageal Carcinogenesis. Int J Oncol (2012) 41(4):1285–96. doi: 10.3892/ijo.2012.1589 22895565

[28] Stepanenko AA Vassetzky YS Kavsan VM. Antagonistic Functional Duality of Cancer Genes. Gene (2013) 529(2):199–207. doi: 10.1016/j.gene.2013.07.047 23933273

[29] Duro E Lundin C Ask K Sanchez-Pulido L MacArtney TJ Toth R. Identification of the MMS22L-TONSL Complex That Promotes Homologous Recombination. Mol Cell (2010) 40(4):632–44. doi: 10.1016/j.molcel.2010.10.023 21055984

[30] O’Connell BC Adamson B Lydeard JR Sowa ME Ciccia A Bredemeyer AL. A Genome-Wide Camptothecin Sensitivity Screen Identifies a Mammalian MMS22L-NFKBIL2 Complex Required for Genomic Stability. Mol Cell (2010) 40(4):645–57. doi: 10.1016/j.molcel.2010.10.022 PMC3006237 21055985

[31] O’Donnell L Panier S Wildenhain J Tkach JM Al-Hakim A Landry MC. The MMS22L-TONSL Complex Mediates Recovery From Replication Stress and Homologous Recombination. Mol Cell (2010) 40(4):619–31. doi: 10.1016/j.molcel.2010.10.024 PMC3031522 21055983

[32] Piwko W Olma MH Held M Bianco JN Pedrioli PG Hofmann K. Rnai-Based Screening Identifies the Mms22L-Nfkbil2 Complex as a Novel Regulator of DNA Replication in Human Cells. EMBO J (2010) 29(24):4210–22. doi: 10.1038/emboj.2010.304 PMC3018799 21113133

[33] Savci-Heijink CD Halfwerk H Koster J van de Vijver MJ. A Novel Gene Expression Signature for Bone Metastasis in Breast Carcinomas. Breast Cancer Res Treat (2016) 156(2):249–59. doi: 10.1007/s10549-016-3741-z PMC4819548 26965286

[34] Welcker M Clurman BE. FBW7 Ubiquitin Ligase: A Tumour Suppressor at the Crossroads of Cell Division, Growth and Differentiation. Nat Rev Cancer (2008) 8(2):83–93. doi: 10.1038/nrc2290 18094723

[35] Lin PC Yeh YM Lin BW Lin SC Chan RH Chen PC. Intratumor Heterogeneity of MYO18A and FBXW7 Variants Impact the Clinical Outcome of Stage III Colorectal Cancer. Front Oncol (2020) 10:588557. doi: 10.3389/fonc.2020.588557 33194745 PMC7658598

[36] Yu H Ling T Shi R Shu Q Li Y Tan Z. [Expression of FBXW7 in Esophageal Squamous Cell Carcinoma and Its Clinical Significance]. Zhonghua Zhong Liu Za Zhi (2015) 37(5):347–51. 26463024

[37] Yokobori T Mimori K Iwatsuki M Ishii H Tanaka F Sato T. Copy Number Loss of FBXW7 is Related to Gene Expression and Poor Prognosis in Esophageal Squamous Cell Carcinoma. Int J Oncol (2012) 41(1):253–9. doi: 10.3892/ijo.2012.1436 22576686

[38] Mlecnik B Bindea G Kirilovsky A Angell HK Obenauf AC Tosolini M. The Tumor Microenvironment and Immunoscore Are Critical Determinants of Dissemination to Distant Metastasis. Sci Transl Med (2016) 8(327):327ra26. doi: 10.1126/scitranslmed.aad6352 26912905

