# Supplementary figures and images for: MMS22L Expression as a Predictive Biomarker for the Efficacy of Neoadjuvant Chemoradiotherapy in Oesophageal Squamous Cell Carcinoma
Source: Front Oncol. 2021 Sep 30;11:711642. doi: 10.3389/fonc.2021.711642 (PMC8514954; doi:10.3389/fonc.2021.711642)

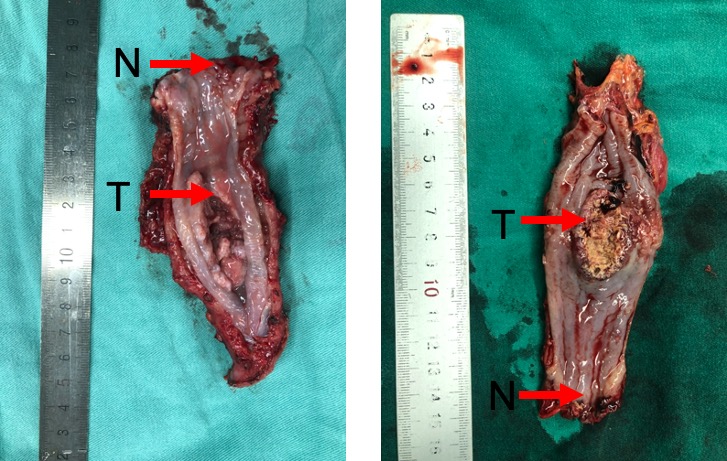

Supplement: Supplementary file 1 [file Image_1.jpeg]
